# Supplementary material for: Preparation, Optimization and In-Vitro Evaluation of Curcumin-Loaded Niosome@calcium Alginate Nanocarrier as a New Approach for Breast Cancer Treatment
Source: Biology (Basel). 2021 Feb 26;10(3):173. doi: 10.3390/biology10030173 (PMC7996962; doi:10.3390/biology10030173)
Supplement: Supplementary file 1 [file biology-10-00173-s001.pdf]

Supporting Information

Preparation, optimization and in-vitro evaluation of curcumin loaded into niosome@calcium alginate nanocarrier as a new approach for breast cancer treatment

Iman Akbarzadeh [a, *], Mona Shayan[b] , Mahsa Bourbour[c], Maryam Moghtaderi[d], Hassan Noorbazargan[e] , Faten Eshrati Yeganeh[f], Samaneh Saffar[a], Mohammadreza Tahriri [g, *]

[a]Department of Chemical and Petrochemical Engineering, Sharif University of Technology, Tehran, Iran.
[b]Core Facility Center, Pasteur Institute of Iran, Tehran, Iran.
[c]Department of Biotechnology, Alzahra University, Tehran, Iran.
[d]Department of Chemical Engineering, Faculty of Engineering, University of Tehran, Tehran, Iran.
[e]Department of Biotechnology, School of Advanced Technologies in Medicine, Shahid Beheshti University of Medical Sciences, Tehran, Iran.
[f] Department of Chemistry, Science and Research Branch, Islamic Azad University, Tehran, Iran.
[g] Department of Developmental Sciences, Marquette University, Milwaukee, WI, 53233, USA.

M.S. and I.A. contributed equally to this work

***Corresponding authors:**
Iman Akbarzadeh, E-mail: Imanakbarzadeh71@yahoo.com

Mohammadreza Tahriri, E-mail: mohammadreza.tahriri@marquette.edu

SI-1. Experimentation

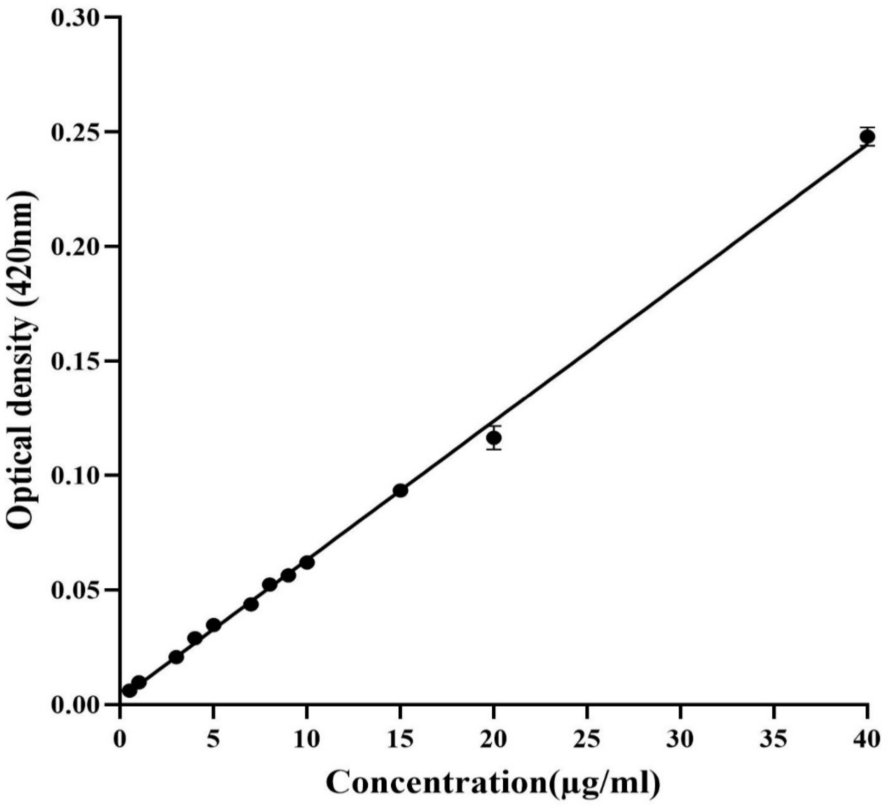

Figure S1. The calibration curve for determination of curcumin based on its maximum absorbance peak at 420 nm in PBS-SDS (0.5%,w/v) solution

SI-2. Kinetic models

The explanation of each kinetic model used in this study is as follow[1]:

- ***Zero-order model:*** $C_t = C_0 + K_0 t$

where C_t represents the amount of drug released at time t, C_0 is the initial concentration of drug released which is generally zero. In this model, the release process takes place at a constant rate and it is independent of initial drug concentration.

- ***First-order model:*** $Log\ C = LogC_0 - Kt/2\cdot 303$

Where C_0 is the initial concentration of the drug, k is the first-order rate constant, and t is the time. C is the drug remaining in the carrier at time t. Log C and t have s linear relationship and K/2.303 is the slope of the straight line. This model can be used to describe water-soluble drugs in porous matrices.

- ***Higuchi model:*** $Q = K_H\sqrt{t}$

where, K_H is the Higuchi constant and it is obtained from the slope of the line. The data obtained were plotted as cumulative percentage drug release versus square root of time. This model can be useful in the case of matrix tablets containing water-soluble drugs.

- ***Korsmeyer-Peppas model:*** $M_t/M_\infty = Kt^n$

Linear Form: logM=Log K+n Logt

where M_t/M_0 is a fraction of drug released at time t, k is the release rate constant and n is the release exponent. The n value is used to characterize different releases for cylindrical shaped matrices. For the case of spherical tablets:

- $n \leq 0.43$: Fickian diffusion mechanism
- $0.43 < n < 0.85$: non-Fickian transport.
- $n = 0.85$: Case II (relaxational) transport.
- $n > 0.85$: super case II transport.

The first 60% drug release data were fitted in this model. Data obtained from drug release studies were

plotted as log cumulative percentage drug release versus log time. This model is suitable for polymeric systems.

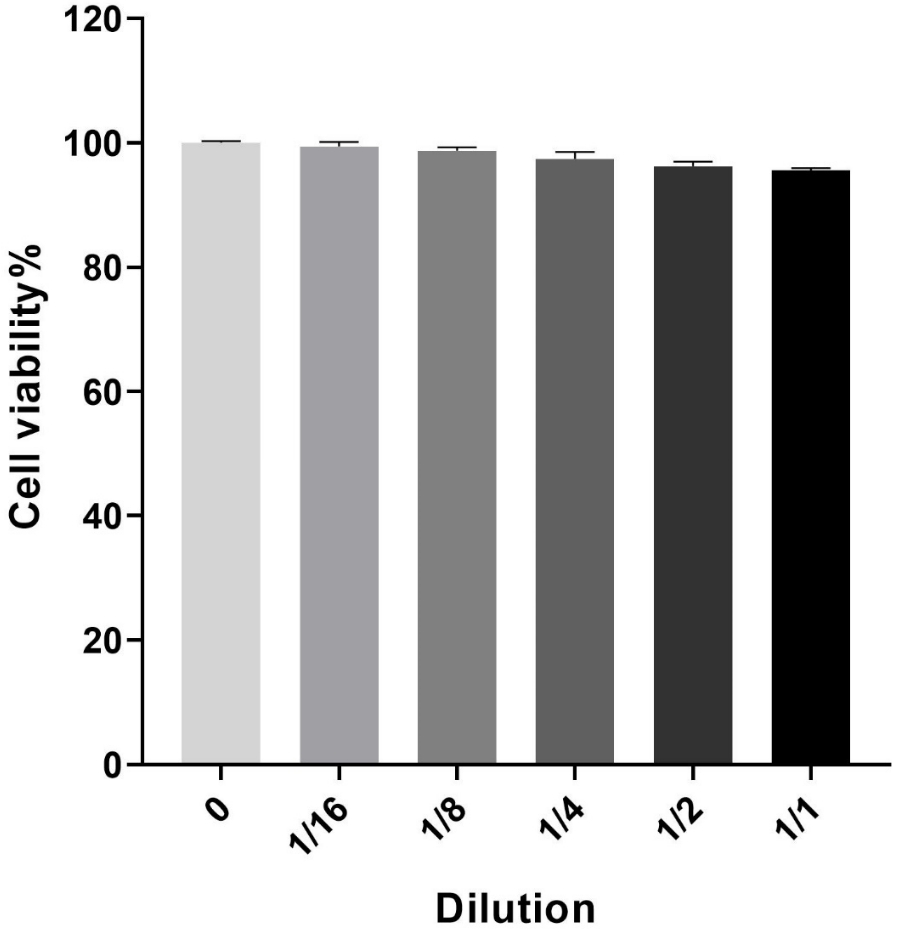

Figure S2. Cell viability of MCF10A cell after 72 h treatment with various dilution of niosomes (Nio).

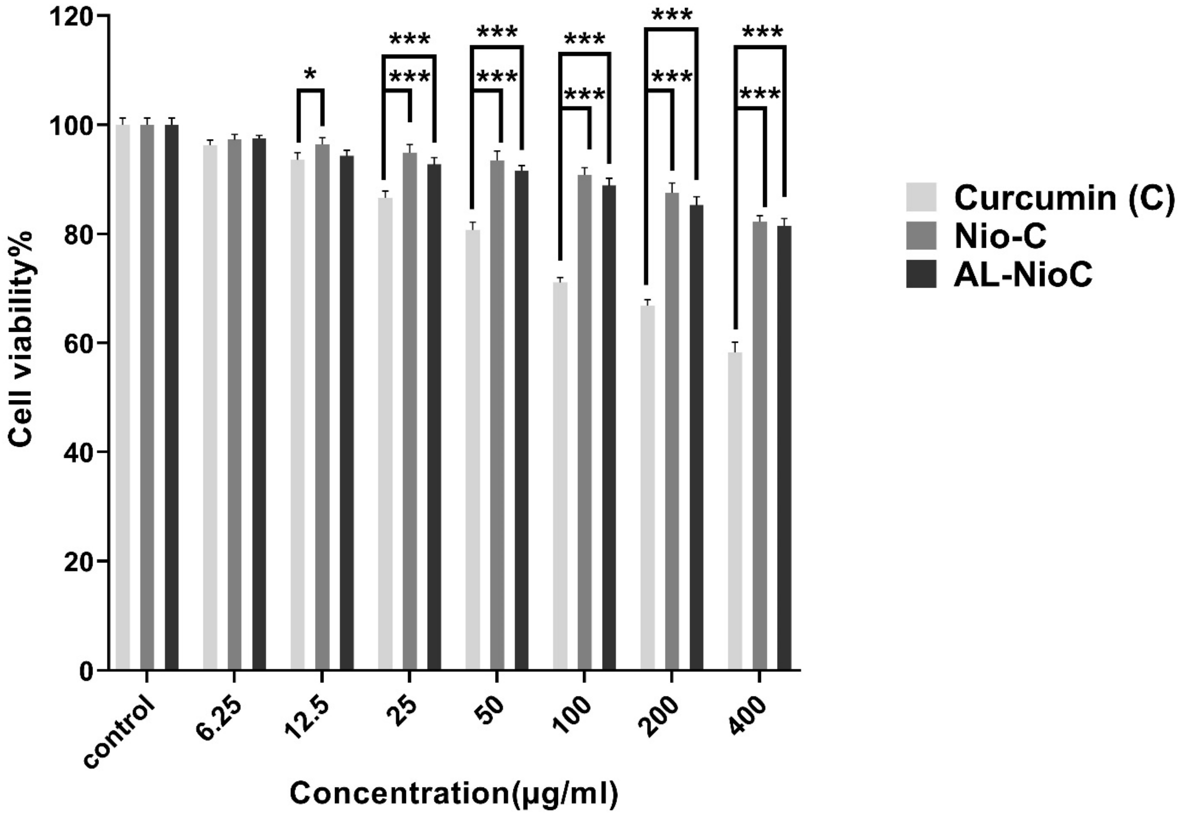

Figure S3. Cell viability of MCF10A cell after 72 h treatment with various concentration of curcumin (C), curcumin loaded niosomes (NioC) and curcumin-loaded niosomes with calcium alginate shell (AL-NioC); Data are represented as Mean ± SD, n = 5. (* $p < 0.05$, and *** $p < 0.001$).

.

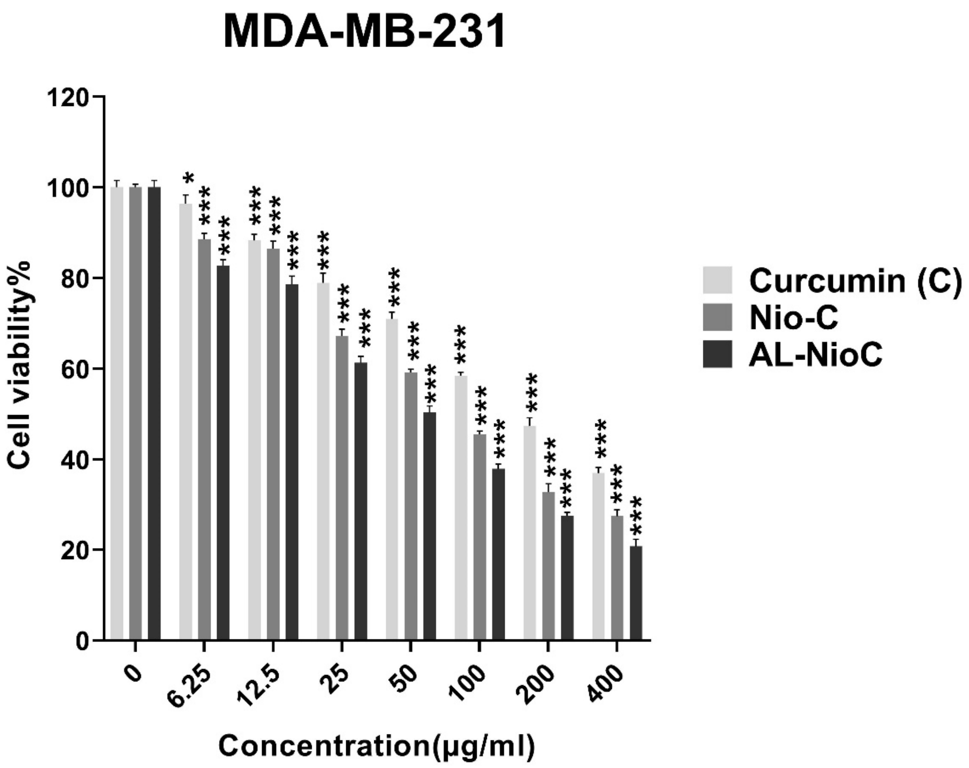
MDA-MB-231
Cell viability%
Concentration(µg/ml)
Curcumin (C)
Nio-C
AL-NioC

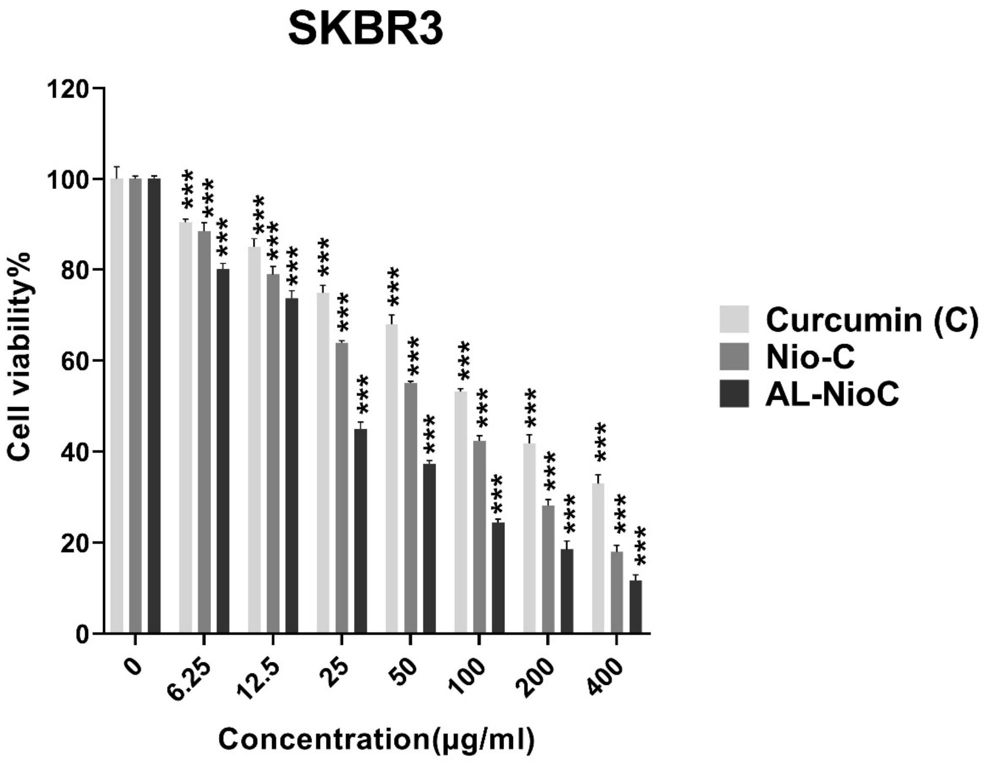
SKBR3
Cell viability%
Concentration(µg/ml)
Curcumin (C)
Nio-C
AL-NioC

Figure S4. Cell viability of MDA-MB-231 and SKBR3 cells after 72 h treatment with various concentrations of curcumin (C), curcumin loaded niosomes (NioC) and curcumin-loaded niosomes with calcium alginate shell (AL-NioC); Data are represented as Mean ± SD, n = 5. (* p <0.05, and *** p <0.001).

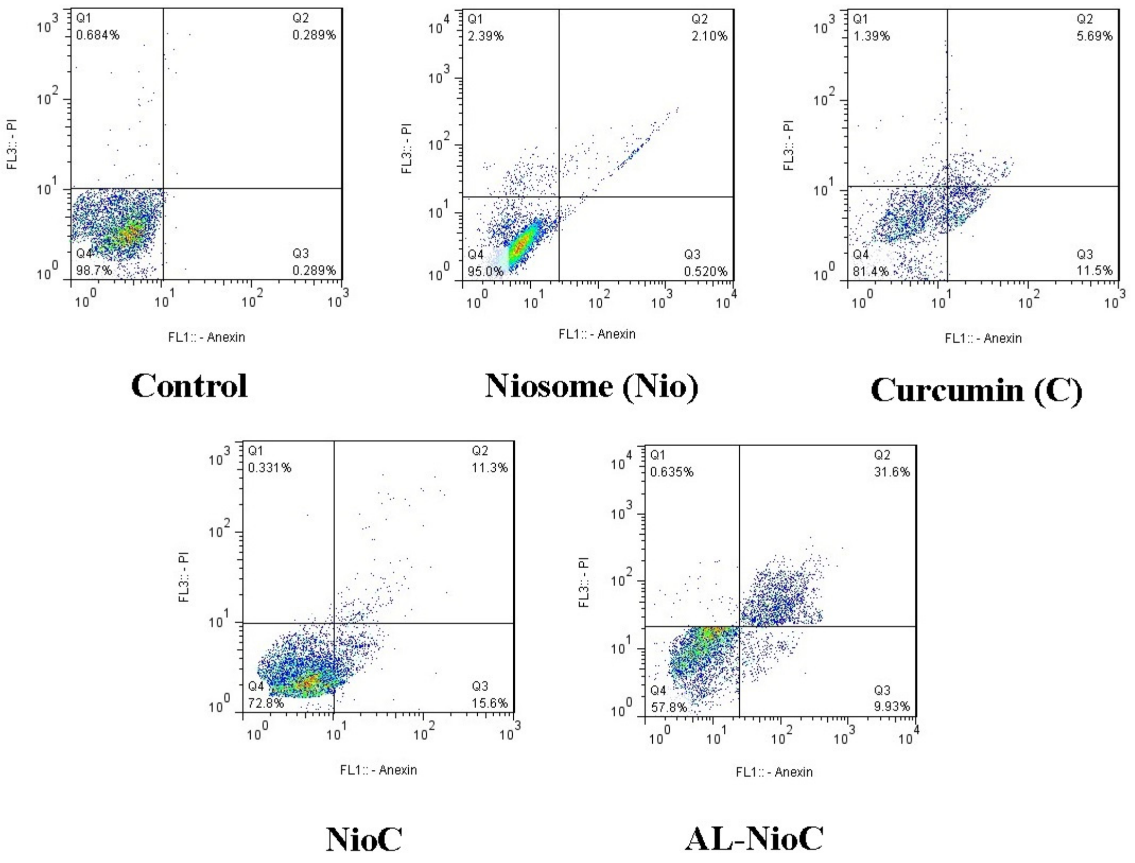

Figure S5. The flow cytometry of MDA-MB-231 cells after treatment with different samples; Lower left panel (Q4): live cells, upper left panel (Q1): necrosis, lower right panel (Q3): early apoptosis, upper right panel (Q2): late apoptosis.

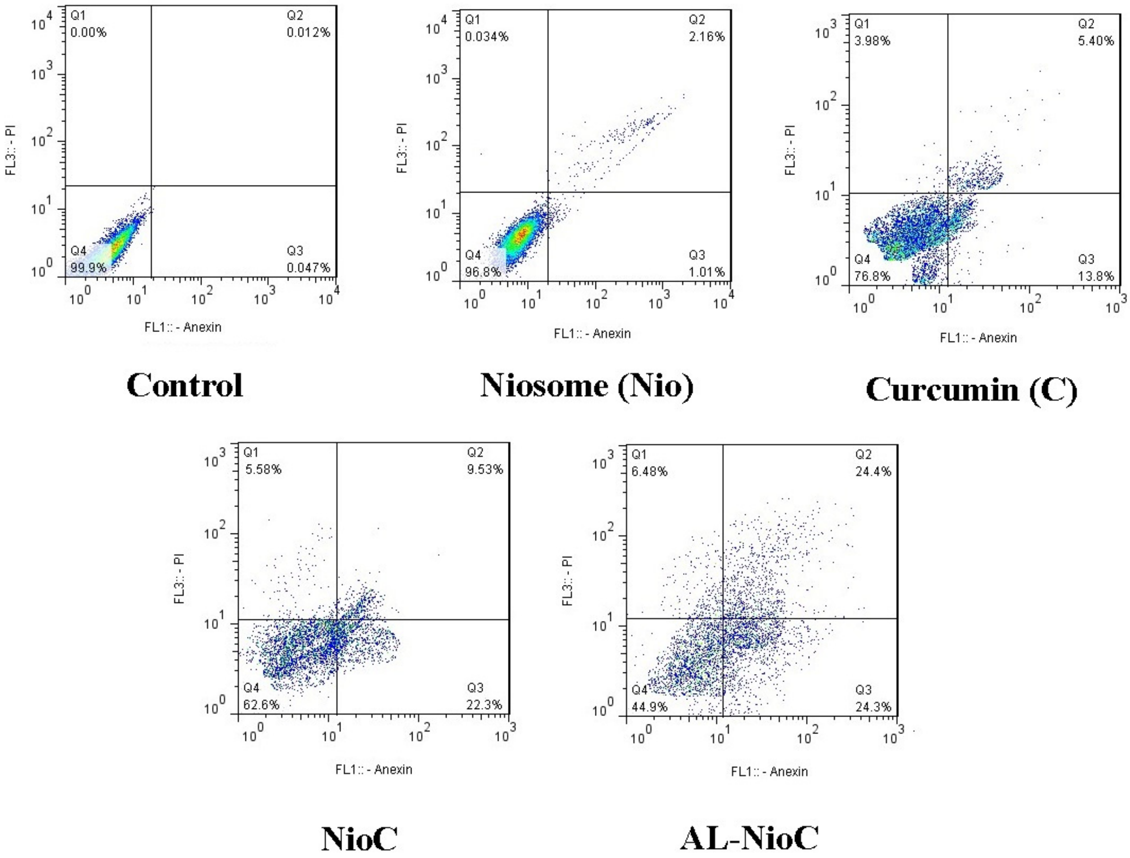

Figure S6. The flow cytometry of SKBR3 cells after treatment with different samples; Lower left panel (Q4): live cells, upper left panel (Q1): necrosis, lower right panel (Q3): early apoptosis, upper right panel (Q2): late apoptosis.

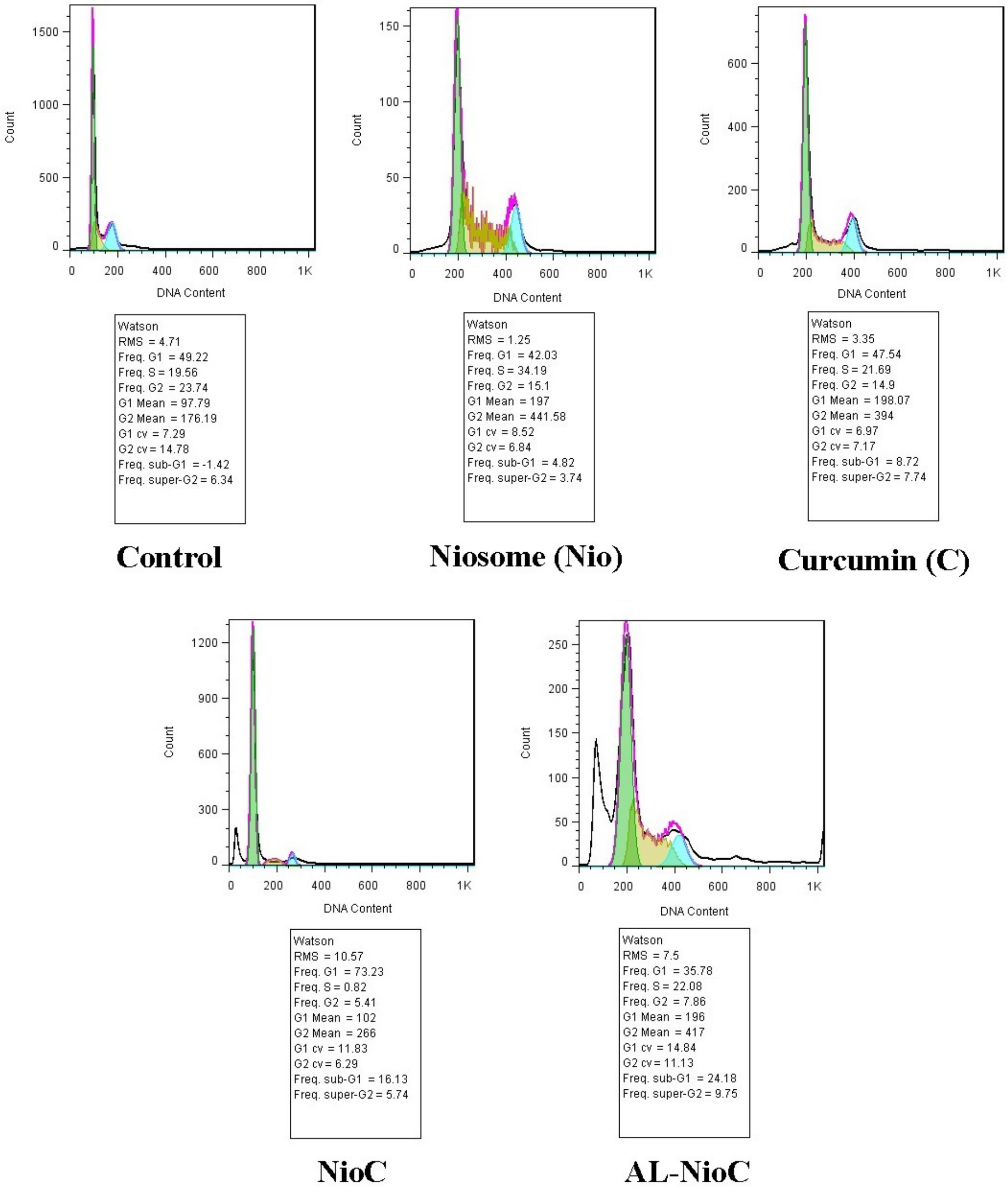

Figure S7. The MDA-MB-231 cell cycle analysis of different samples

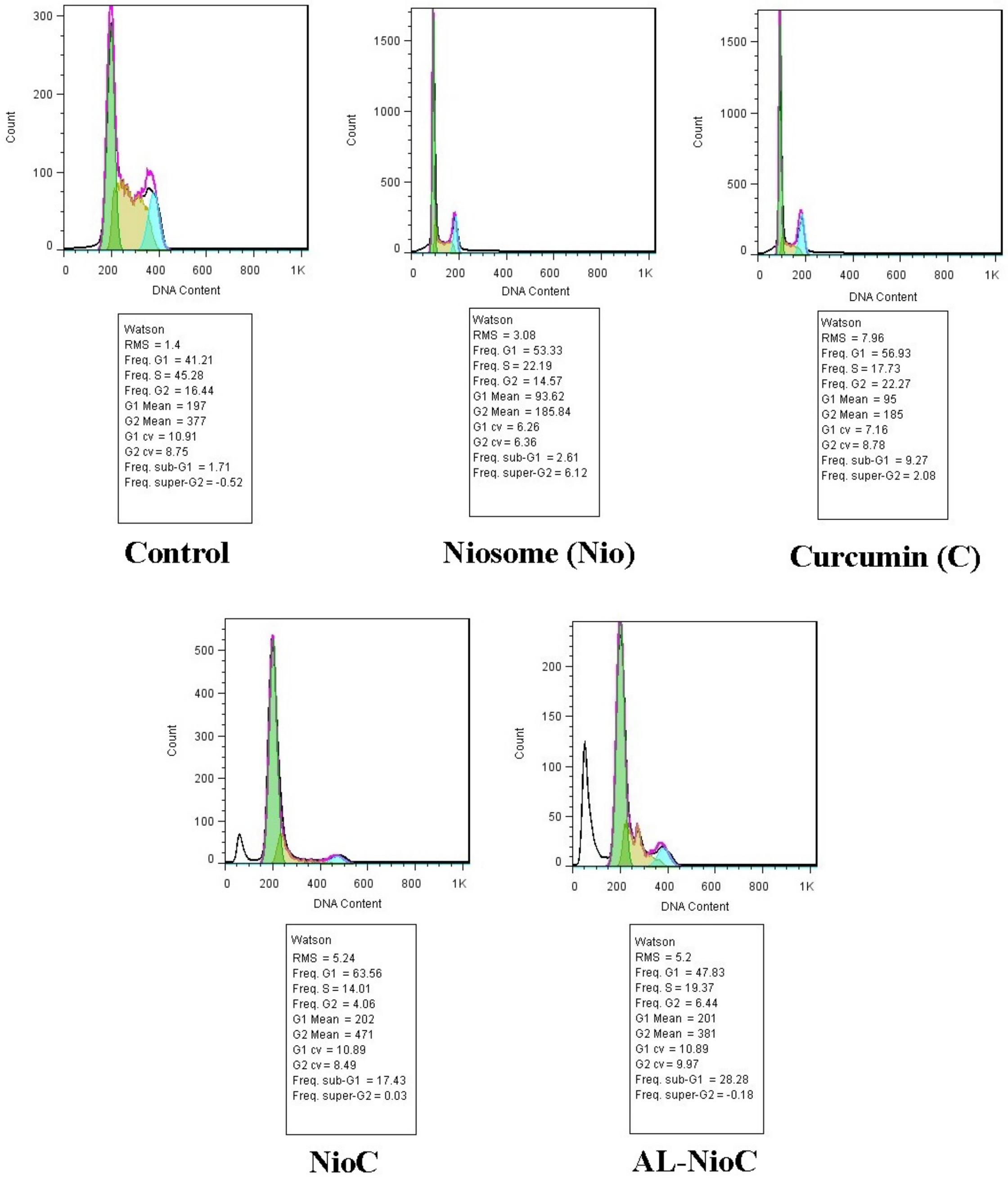

Figure S8. The SKBR3 cell cycle analysis of different samples

Reference:

[1] S. Dash, P.N. Murthy, L. Nath, P. Chowdhury, Acta Pol Pharm, 67 (2010) 217-223.
